# Residential Institutions for Mental Defectives

**Published:** 1912-06-15

**Authors:** 


### THE DEFECTIVE AND DEPENDENT.*

### IX.—Residential Institutions for Mental Defectives.

As may have been gathered from previous papers, the system of instruction of mentally-defective children in special day-schools leaves much to be desired. Coming as most of the pupils do from poor homes, often situated in the crowded slums of great cities amidst unfavourable moral and sanitary surroundings, there is a tendency for much of the good gained by school attendance for four or five hours daily to be effaced in the remainder of the twenty-four. And when the age of sixteen arrives the control of the education authority entirely ceases, so that at the most critical period of life, that of adolescence, the pupil, however improved, has to fight the battle of life handicapped by ineradicable congenital defect with such aid as can be rendered by After-care Committees and other charitable organisations. Failure to maintain the unequal struggle is in a large number of cases the inevitable result, and consequently the time and money lavished in an elaborate training proves to be more or less nugatory. It is to be hoped that legislation now before Parliament may supplement existing arrangements by the establishment, with the aid of public funds, of residential industrial institutions for cases needing permanent care.

What has been done by voluntary agencies in the past?

### Imbecile Institutions.

Voluntary residential institutions for mental defectives of various grades (though originally designated "Asylums for Idiots") have been in existence in this country for more than half a century. Some reference has already been made to them in an early paper of this series (No. III., December 2, 1911). At present they number nine, viz., six in England, two in Scotland, and one in Ireland, and their aggregate accommodation is for about 2,500 inmates. These are all charitable foundations, and for the most part patients are admitted by the votes of subscribers at periodical elections, though a certain number are received at various rates of payment by their relations, and in one or two instances at the cost of Guardians.

The majority were originally intended to be training institutions, and with this view inmates were received only for periods of five or seven years. Experience, however, soon demonstrated the necessity of permanent care in a considerable proportion of cases, and arrangements were made for re-elections for further periods and for a certain number (limited by financial considerations) of life cases. At the present time the older institutions show a large percentage of inmates of long standing: thus at Earlswood 20 per cent. are stated to be life-cases, and 41 per cent. have been in residence over ten years. At the Royal Eastern Counties Institution, Colchester, about 20 per cent. only of the patients admitted since its foundation in 1859 have been permanently discharged, and all the others admitted are still under treatment or have died in the institution. At the Royal Albert Institution, Lancaster, it is stated in the last report that "out of the 704 inmates about two-thirds have been already received for unlimited periods," and an effort is now being made to raise £12,000 for the purpose of providing colony homes on the institution estate for the permanent care of all whose discharge is considered undesirable. It would appear, therefore, that the voluntary institutions which were earliest in the field in the work of ameliorating the condition of all classes of the congenitally-defective (including those now designated feeble-minded, as well as idiots and imbeciles) are at present fully alive to the urgency of providing not only training but permanent care, and it is to be hoped that the State will supplement, rather than supersede, their beneficent and experienced, if circumscribed, activities.

### Industrial Homes for Feeble-minded.

During the closing decade of the last century a widespread interest arose amongst philanthropic persons in the large class who in popular language are called "Feeble-minded," that is to say persons so deficient in serviceable common sense and self-directing power as to be incapable of managing themselves and their affairs with ordinary prudence. Under favourable circumstances such may indeed become partially, if not entirely, self-supporting; and it is to supply these favourable circumstances, with suitable employment, that the comparatively recent establishment of industrial homes for feeble-minded young persons has been brought about. Since 1890 nearly thirty small residential homes have been organised in various parts of the country, the majority being for girls and young women, who specially need the protection from moral harm which they afford. The predominant occupation in these homes is laundry-work, from which a considerable portion of the necessary income is derived. Poor-Law cases are taken at rates varying from 8s. to 10s. a week, and inmates are also received on payment from parents. It has been the aim of the ladies who have founded these homes to conduct them as far as possible on a family basis, and in general the number of inmates varies from twenty to thirty. In addition to laundry-work, domestic work, needlework, rug-making and basket-making are taught, and, in a few, girls are trained for domestic service, though experience shows that satisfactory situations for such girls are not easily found. Indeed the tendency is to organise the majority of these homes for permanent care. At a home for girls near Liverpool horticulture has recently been adopted as an occupation for the inmates, and this and farm work, poultry farming, etc., form the staple employment of the comparatively few establishments existing for boys and young men. At the present time accommodation for about 2,000 inmates has been provided by voluntary effort in residential homes and colonies for the feeble-minded, and of the colonies we shall speak later.

* Previous articles appeared on Oct. 7, 21, Nov. 18, Dec. 2, Jan. 6, Feb. 10, 17, March 2, 16, 23, May 4.

